# Fecal DNA Identifies a Disjunct Population of an Endemic Deer (*Mazama jucunda*) From the Atlantic Forest, Brazil

**DOI:** 10.1002/age.70134

**Published:** 2026-06-05

**Authors:** Jeferson L. S. Freitas, Pedro H. F. Peres, Francisco Grotta‐Neto, Márcio L. Oliveira, José M. B. Duarte

**Affiliations:** ^1^ Deer Research and Conservation Center (NUPECCE) São Paulo State University (UNESP) Jaboticabal São Paulo Brazil; ^2^ Evolutionary Genetics and Molecular Biology Graduate Program Federal University of São Carlos (UFSCar) São Carlos São Paulo Brazil; ^3^ Department of Biological Sciences and Health University of Araraquara (UNIARA) Araraquara São Paulo Brazil

**Keywords:** Atlantic forest, brocket deer, evolutionarily significant unit, fecal DNA

## Abstract

Limited knowledge of brocket deer distribution persists due to their elusive behavior and the morphological similarities among species. A population of red brocket deer (genus *Mazama*) was recently discovered in the Rio Doce State Park (PERD), a protected area within the Brazilian Atlantic Forest; however, species‐level identification was lacking. Given that two endemic and threatened red brocket species inhabit this biome, we aimed to identify the species present in PERD using fecal DNA analyses. We sequenced six mitochondrial DNA regions (1450 bp) from fecal samples collected in PERD and other Atlantic Forest sites, analyzing them against reference specimens. We conducted phylogenetic analyses and employed coalescent‐based molecular species delimitation methods (GMYC and bPTP) to define molecular operational taxonomic units (MOTUs). Additionally, we constructed a haplotype network and a genetic distance matrix. Our analyses linked the PERD samples to *Mazama jucunda* (small red brocket) but revealed a reciprocally monophyletic topology, identifying two distinct MOTUs. PERD harbors unique haplotypes, and its genetic distance to *M. jucunda* is comparable to that between *M. jucunda* and *M. nana* (Brazilian dwarf brocket). We propose that PERD hosts an evolutionarily significant unit (ESU) of *M. jucunda* , located 700 km from its known range. The observed genetic structure and PERD's isolation support the hypothesis of a potentially new red brocket species, warranting further cytogenetic investigation. This study represents a significant advancement in understanding the distribution and genetic structure of a threatened Neotropical forest deer and has immediate conservation implications in one of the world's most degraded biodiversity hotspots.

## 1 Introduction

The Atlantic Forest is one of the most biodiverse and threatened tropical forests in the world. The high levels of endemism (Mittermeier et al. 2011) and the severe loss of approximately 88% of its original coverage (Ribeiro et al. 2009) make it one of the world's most important hotspots for biodiversity conservation (Myers et al. 2000). Ungulates are key species in neotropical forests due to their various ecological roles, such as soil displacement, herbivory, seed dispersal, and as prey for large predators (Peres 2000; Beck 2006; Beck et al. 2010, 2013). Among them, brocket deer have evolved under strong selective pressures to adapt to dense forests (Oliveira et al. 2025) and are considered indicators of forest preservation due to their ecological demands for pristine areas (Duarte et al. 2017; Oliveira et al. 2019). In the Atlantic Forest, three red brocket deer species (genus *Mazama*) are found: *M. rufa* , *M. nana* , and *M. jucunda* (Duarte et al. 2017; Oliveira et al. 2019; Peres, Grotta‐Neto, et al. 2021). Both *M. nana* and *M. jucunda* are endemic to this biome and are currently classified as Vulnerable by the International Union for Conservation of Nature (IUCN) (Duarte et al. 2015; Vogliotti et al. 2016). Moreover, *M. jucunda* is not only a threatened and endemic species (the largest endemic mammal in the Atlantic Forest) but it has one of the most restricted distributions among deer species in the world (Weber and Gonzalez 2003). Despite their ecological significance, these species face severe threats, including poaching, habitat loss, disease transmission from domestic livestock, and predation by domestic dogs (Duarte et al. 2015; Duarte and Vogliotti 2015; Vogliotti et al. 2016).

In addition to these threats, the conservation of *Mazama* species is further hindered by the historical presence of significant taxonomic gaps (González and Duarte 2020). These gaps are mostly related to the fact that these animals are extremely elusive and still have a high degree of homoplasy in morphological characters while being different molecularly and cytogenetically (Duarte et al. 2008; Peres, Luduvério, et al. 2021; Bernegossi et al. 2024). For example, cryptic species complexes have been identified—cases in which two or more distinct species are mistakenly classified under a single species name (Bickford et al. 2007). This is the case of the species *M. americana* (Duarte et al. 2008; Gutiérrez et al. 2017), for which different chromosomal patterns have been identified, referred to as cytotypes ( *M. americana* sensu lato), with geographical coherence throughout the distribution of *M. americana* (sensu lato) in Brazil (Bernegossi et al. 2024). Recently, Cifuentes‐Rincón et al. (2020) indicated a neotype for the species, thus fixing its chromosomal pattern ( *M. americana* sensu stricto). In addition to the taxonomic issues, information on the geographical distribution of these species is still scarce; only *M. jucunda* and *M. nana* have had their areas of occurrence modeled in studies carried out specifically with this purpose (Duarte et al. 2017; Oliveira et al. 2019).

**FIGURE 1:**
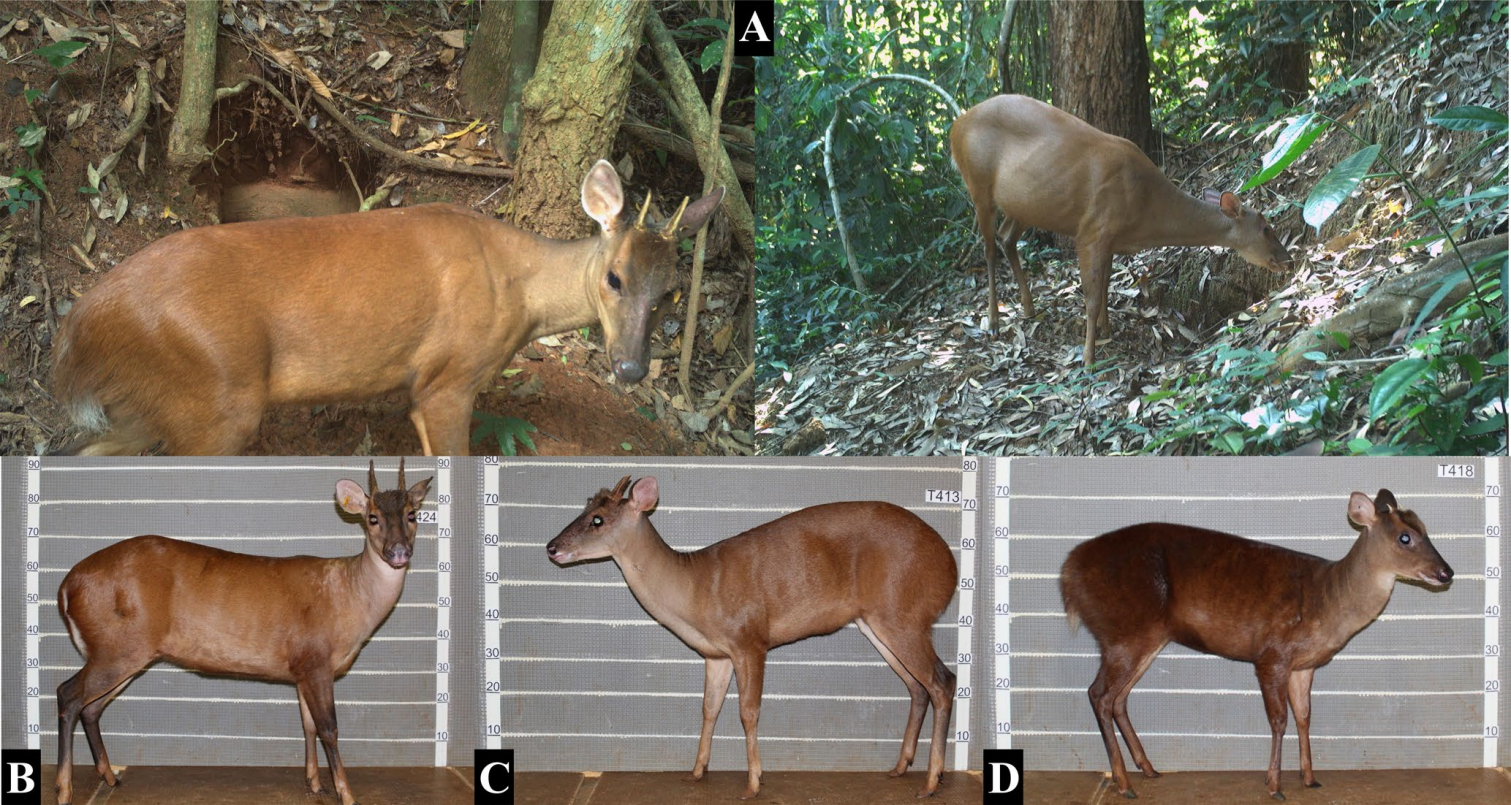
Individuals of Rio Doce State Park recorded by camera traps (A) and species of the genus *Mazama* with similar size and external morphology (B = *Mazama americana* , C = *Mazama rufa* and D = *Mazama jucunda*). Scale in centimeters. *Source:* Wild Animal Conservation Institute (ICAS; images in A) and Deer Research and Conservation Center (NUPECCE; images in B, C, and D).

The forest habitats, often dense and difficult to access, and the elusive behavior of these species make it very costly to capture individuals to obtain morphological data and live cells to perform cytogenetic analyses, which are very informative in the taxonomic resolution of the group (Cifuentes‐Rincón et al. 2020; Peres, Luduvério, et al. 2021; Sandoval et al. 2022; Bernegossi et al. 2023; Morales‐Donoso et al. 2023). In this context, non‐invasive sampling methods are particularly valuable. Camera traps can assist in the identification of recorded species and provide ecological data (Grotta‐Neto et al. 2020). The identification of forest deer by this method, however, is limited due to the extreme similarity, and very similar color patterns, as is the case with *M. americana* , *M. rufa* , and *M. jucunda* (Figure 1; Peres, Luduvério, et al. 2021). Another widely used methodology is fecal sampling followed by species‐level genetic identification using mitochondrial DNA markers (Oliveira et al. 2022). This methodology also allows estimates of population density and geographic distribution modeling (Duarte et al. 2017; Oliveira et al. 2019; Silva et al. 2020; Morini et al. 2024).

In the context of the Atlantic Forest, an important gap regarding the geographical distribution of red brocket deer is the identification of species inhabiting the Rio Doce State Park (PERD), one of the largest continuous areas of this biome (Ribeiro et al. 2009). Using fecal DNA, Oliveira et al. (2020) performed phylogenetic analysis with fragments of the mitochondrial *cytochrome b (CYTB)* gene and demonstrated that this population belongs to the genus *Mazama*, but they were not able to carry out the identification at the species level because the mitochondrial marker used was not sufficiently informative, being necessary to add more polymorphic regions (Peres, Grotta‐Neto, et al. 2021). Moreover, even though the authors had access to images of the animal in the area (Figure 1), it was not possible to perform the identification due to high morphological similarity to other closely related species (Peres, Luduvério, et al. 2021). In this context, we aimed to identify the deer species inhabiting the PERD using fecal DNA genetic analysis and a comprehensive sampling of potential species.

## 2 Materials and Methods

### 2.1 Study Area

The Rio Doce State Park (PERD) is the main continuous area of Atlantic Forest in the state of Minas Gerais covering 35 976 ha. The predominant vegetation typology in the area is the Submontane Semi‐deciduous Seasonal Forest. The fauna of PERD is very diverse, including taxa endemic to the Atlantic Forest and several species threatened with extinction, such as the northern muriqui ( *Brachyteles hypoxanthus* ), the brown howler monkey ( *Alouatta guariba* ), the giant armadillo ( *Priodontes maximus* ), and the jaguar ( *Panthera onca* ), and other ungulates, such as the lowland tapir ( *Tapirus terrestris* ). Although the park's Management Plan (IEF 2023) indicates the presence of only one deer species, the red brocket deer ( *Mazama americana* ), there are also indications of the presence of another forest deer, the gray brocket deer (*Subulo gouazoubira*), whose presence within the unit remains to be confirmed.

### 2.2 Fecal and Reference Samples

We collected fecal samples using a scat detection dog specially trained to locate feces of all Brazilian deer species, conditioned to not interact with wildlife, and subjected to strict sanitary control. The searches took place along 20 transects, each up to 1 km long, throughout the entire park. The dog searched for samples freely, without a leash, moving within a maximum radius of 20 m around the handler. Each sample was georeferenced and identified with the acronym PERDJ, collected without direct hand contact, and stored in 50 mL plastic tubes containing absolute ethanol. Our survey encompassed approximately 24 km, over 40 h of search effort, yielding a total of 108 fecal piles collected (4.5 samples/km and 2.7 samples/h).

We also included in this study samples previously collected and identified at the species level, deposited in the Fecal Bank of the Deer Research and Conservation Center (NUPECCE) of the São Paulo State University (UNESP). Some of these samples are from the PERD (Oliveira et al. 2020) and the remaining of them are from other protected areas in the states of São Paulo, Paraná, and Santa Catarina (Oliveira 2015; Grotta‐Neto et al. 2024).

We included a reference dataset composed of *Mazama* specimens with reliable identification provided by morphological and cytogenetic evaluation. All specimens were from the database of samples and sequences managed by NUPECCE. We selected a total of 36 samples, including two *M. americana* sensu stricto, five *M. jucunda* , eight *M. nana* , four *M. rufa* , and three *M. temama* . We also included individuals from *M. americana* sensu lato cytotypes: Acre (three samples), Carajás (four samples), Juína (three samples), and Rondônia (four samples).

### 2.3 DNA Extraction, Amplification, and Sequencing

We conducted all laboratory procedures involving fecal samples in a dedicated room for fecal DNA analysis to prevent cross‐contamination. We extracted fecal DNA using a modified silica‐based protocol described by Höss and Pääbo (1993) originally developed for ancient bone samples. Approximately 1 mL of extraction buffer was added to homogenized fecal material and incubated at 60°C for 1 h with occasional manual mixing. After centrifugation, about 500 μL of the supernatant was combined with 500 μL of fresh extraction buffer and 40 μL of silica suspension to bind the DNA. Following a 10‐min incubation at room temperature, the mixture was centrifuged, the supernatant was discarded, and the silica‐bound DNA was washed twice with 1 mL of wash buffer and then twice with ethanol (70% and 100%). After drying, DNA was eluted in TE buffer and incubated overnight at 55°C to maximize recovery. The eluted DNA was then purified through two additional centrifugation steps to remove any residual silica particles. NUPECCE bank supplied DNA from prior tissue extractions for the reference samples that required sequencing.

First, we screened all fecal samples to exclude samples of *Subulo gouazoubira* using a PCR‐RFLP protocol based on a 224 bp fragment of the mitochondrial *CYTB* gene (González et al. 2009). For full amplification and sequencing of *M. jucunda* samples, we selected five spatially distributed samples from PERD and six additional fecal samples of *M. jucunda* from other protected areas across the species' range in the Atlantic Forest. We performed both fecal and reference sample DNA amplification by PCR using primers designed to amplify fragments (149 to 463 base pairs) of the mitochondrial genes *CYTB*, *NADH dehydrogenase* subunit 5 and 2 (*ND5* and *ND2*), and *cytochrome c oxidase* subunit 1 (*COI*) (Table S1). The PCR reactions were standardized to a final volume of 30 μL (25 μL reaction mix and 5 μL DNA), containing 1× buffer and 1.5 U of *Taq* DNA polymerase (both Platinum, Thermo Fisher Scientific); 2 mM MgCl_2_; 0.6 mM dNTP; 1.3 mg/mL BSA and 0.5 pM of each primer. The reactions were performed in a Bio‐Rad C1000 Touch Thermal Cycler in a touch‐down protocol, which totaled 45 cycles under the following conditions: an initial denaturation step at 94°C for 2 min; followed by 5 cycles of 94°C‐50 s/58°C‐50 s/72°C‐50 s; 6 cycles of 94°C‐50 s/57°C‐45 s/72°C‐50 s; 8 cycles of 94°C‐50 s/56°C‐40 s/72°C‐50 s; 13 cycles of 94°C‐50 s/55°C‐35 s/72°C‐50 s; 13 cycles of 94°C‐50 s/54°C‐30 s/72°C‐50 s and finally a final extension at 72°C for 10 min.

Finally, we visually confirmed the PCR products by electrophoresis on a 2% agarose gel, purified them using the Wizard SV Gel and PCR Clean‐Up System (Promega), and sequenced them (forward and reverse) using the Sanger method on an ABI 3500 Genetic Analyzer (Applied Biosystems). We reviewed the resulting electropherograms, removed the primers, and generated the consensus sequences in BioEdit 7.2.5 (Hall 1999). Except for three reference animals (T021, T064, and T002) that required Sanger sequencing for two gene fragments, we extracted mtDNA regions of interest from mitogenomes deposited in the NUPECCE bank. Details on the mitogenome assembly are described in Bernegossi et al. (2023). We deposited all produced and extracted sequences in GenBank (Tables S2 and S3).

### 2.4 Characterization of the DNA Matrix

**FIGURE 2:**
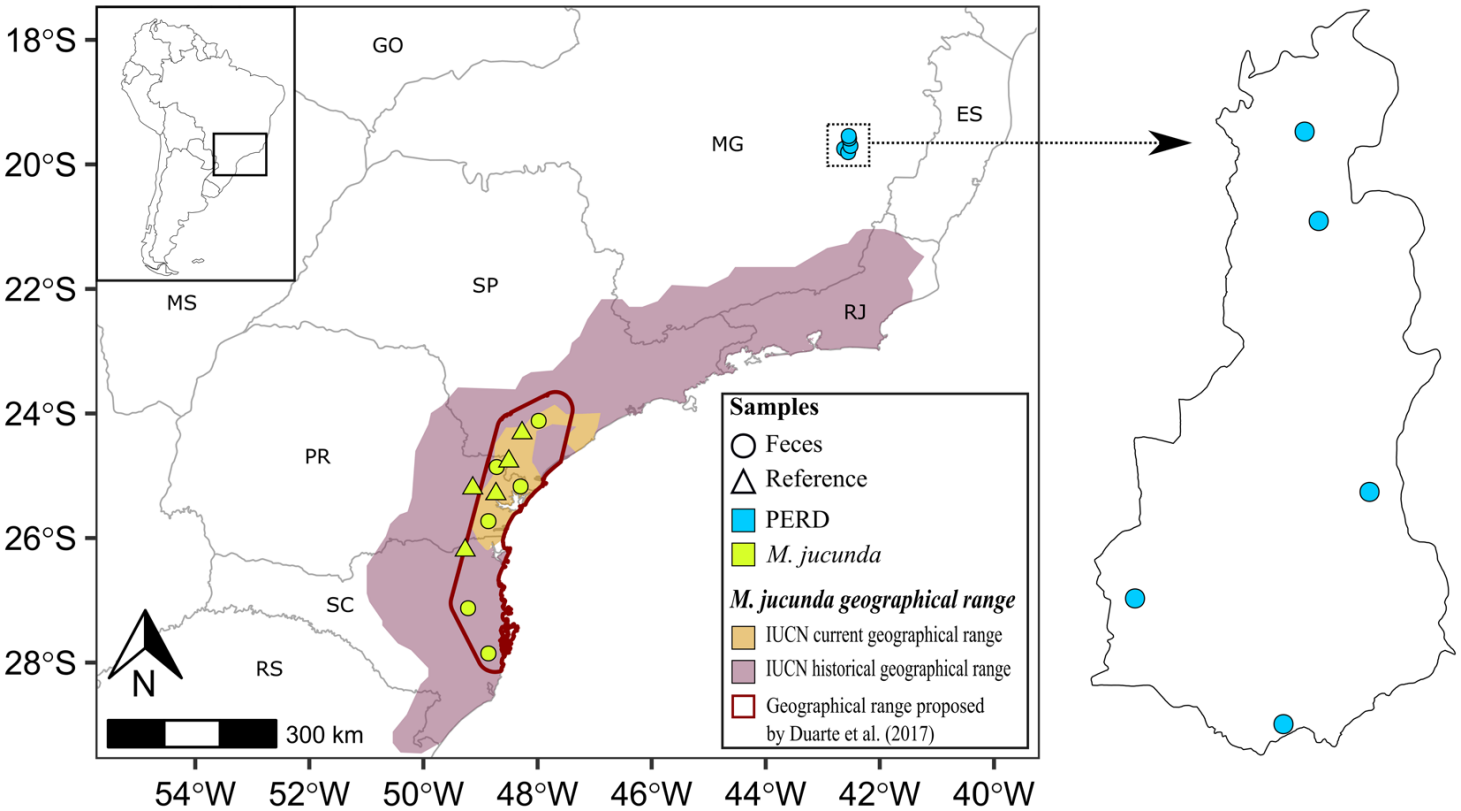
Fecal samples from Rio Doce State Park (PERD) and reference samples of *Mazama jucunda* included in this study.

We added two extra reference samples from GenBank, one *M. americana* sensu stricto, one *M. temama* , and three other species of the family Cervidae comprising the outgroup in the phylogenetic analysis, *Alces alces, Capreolus pygargus*, and *Rangifer tarandus* . The final dataset was composed of 36 reference samples from this study, 5 reference samples from GenBank, and 11 fecal samples, five of which were from PERD (Tables S2 and S3). The location of all samples included in this study can be seen in Figure S1. The locations of the PERD fecal samples and *M. jucunda* reference samples included in this study are shown in Figure 2.

We grouped the samples sequenced in this study together with the reference samples by gene fragment and aligned them using the MAFFT online server (Katoh et al. 2019; https://mafft.cbrc.jp/alignment/server/). We reviewed and edited the alignments in BioEdit and concatenated them in Mesquite 2.75 (Maddison and Maddison 2023), thus forming the final matrix of 1450 bp. We analyzed the matrix polymorphism in MEGA 11 (Tamura et al. 2021) by counting the variable sites and parsimony‐informative characters (PICs—sites with mutations shared by at least two samples). Finally, we used MrModelTest 2.4 (Nylander 2004) to estimate the best‐fitting evolutionary model, selecting the one with the lowest AIC value among the 24 models tested.

### 2.5 Phylogenetic Analysis

We performed the phylogenetic analyses using Bayesian Inference (BI) using the BEAST 1.10.4 package (Suchard et al. 2018). We set the run parameters in the program BEAUti (BEAST package) and implemented the analyses in three different runs on the CIPRES Science Gateway online server (Miller et al. 2010). Each run consisted of four Markov Chain Monte Carlo (MCMC) chains with 50 million generations and with sampling every 1000 generations. We implemented a lognormal uncorrelated relaxed clock and the Yule process speciation tree model. We combined the results of the three independent runs in the Log Combiner program (BEAST package) by applying a *burn‐in* of 25% and confirmed convergence of the analysis using Tracer 1.7.2 software (Rambaut et al. 2018), which was considered satisfactory when the Estimated Sample Size (ESS) exceeded 200. We visualized and graphically modified the final tree using FigTree 1.4.4 (http://tree.bio.ed.ac.uk/software/figtree/), incorporating the posterior probability (PP) as node support derived from the Tree Annotator program within the BEAST package.

In addition to the ultrametric phylogenetic tree generated in BEAST, we generated a non‐ultrametric tree in the program MrBayes 3.2.7a (Ronquist et al. 2012) to be used as input in the bPTP molecular species delimitation method (see below). This analysis was also performed on the CIPRES Science Gateway online server, and was composed of two independent runs with four MCMC chains and 30 million generations, sampled every 1000 generations and combined after a 25% burn‐in. Convergence was verified when the final average standard deviation of split frequencies parameter reached a value below 0.01.

### 2.6 Molecular Species Delimitation

We performed two molecular species delimitation approaches to identify MOTUs (molecular operational taxonomic units), GMYC (General Mixed Yule Coalescent; Fujisawa and Barraclough 2013) and bPTP (Bayesian implementation of the Poisson Tree Process; Zhang et al. 2013). We conducted the GMYC analysis using the splits package 1.0 (Ezard et al. 2021) in R 4.2.2 (R Core Team 2024). We applied the ultrametric phylogenetic tree generated in BEAST and tested two delimitation thresholds (single‐ and multiple‐thresholds). The probabilities of the GMYC models were compared against a null model (assuming a single MOTU), considering results statistically significant when *p* < 0.05. We performed the bPTP analysis on its dedicated web server (https://species.h‐its.org/), using both the ultrametric tree (BEAST) and the non‐ultrametric tree (MrBayes). In total, four species delimitation hypotheses were evaluated: (1) GMYC single‐threshold, (2) GMYC multiple‐threshold (both ultrametric), (3) bPTP with the non‐ultrametric tree, and (4) bPTP with the ultrametric tree.

For both analyses, we removed the outgroups from the phylogenetic trees prior to species delimitation, since outgroups can impact the accuracy of results in tree‐based delimitation methods. Both GMYC and bPTP are designed to detect the transition from interspecific (speciation) to intraspecific (coalescent) branching within a group. The presence of distant outgroups can artificially lengthen branches and distort the transition between inter‐ and intraspecific branching, reducing delimitation accuracy (Fujisawa and Barraclough 2013; Zhang et al. 2013; Luo et al. 2018; Magoga et al. 2021).

### 2.7 Haplotype Network and Genetic Distance

For both analyses, we used the final matrix excluding the outgroup, with sequences grouped either by their presumed species (for reference samples and previously identified fecal samples) or designated as PERD (for PERD fecal samples). We constructed a median‐joining haplotype network in PopART 1.7 (Leigh and Bryant 2015) and estimated a matrix of pairwise mean genetic distances among clusters using the Kimura 2‐parameter (K2P; Kimura 1980) model in MEGA 11 for comparison.

## 3 Results

### 3.1 Fecal Samples Collection and Screening

From 108 fecal samples collected, five were identified as *S. gouazoubira* (PERDJ51, 54, 55, 56, and 58), three failed PCR amplification (PERDJ49, 62, and 75), and 100 were presumed to belong to *M. jucunda* and were retained for sequencing. A prior study by our research group (Oliveira et al. 2020) collected 12 samples in PERD, five of which (PERD2, 4, 6, 7, and 11) were phylogenetically assigned to the genus *Mazama* using mitochondrial *CYTB* fragments. Based on spatial distribution and sequence availability, five samples were selected for the final matrix: PERD2, PERD4, and PERD7 (Oliveira et al. 2020), and PERDJ12 and PERD17 (this study; Figure 2). It is worth mentioning that the complete PCR‐RFLP protocol would theoretically allow the identification of *M. jucunda* samples (González et al. 2009). However, when analyzing the sequences of PERD samples—produced by Oliveira et al. (2020) and in this study—we observed a mutation in the restriction site of the enzyme BstN, which is used to discriminate *M. jucunda* from *M. nana* . This mutation prevented identification at the species level (data not shown). Fortunately, the distribution of *M. nana* is restricted to the southern regions of Brazil and this issue did not affect the identification of PERD samples.

### 3.2 Sequencing and DNA Matrix Characterization

We successfully sequenced all selected fecal samples (*n* = 11) and reference samples requiring sequencing (*n* = 3; T021, T064, T002) to complete the gene fragments in the matrix. The only exception was the fragment of the *COI* gene for the fecal sample PERDJ17. For this sample and sequence, the corresponding region in the matrix was filled with the ambiguity symbol “N”. The final matrix consisted of 52 samples (including three outgroup samples) and was 1450 bp in length, of which 395 were variable sites and 258 were parsimony‐informative characters (PICs) (Table 1). The evolutionary model selected for the final matrix was GTR + G.

**TABLE 1:** Size and polymorphism of the final matrix and the five fragments that compose it.

Region	Size*	Variable sites	PICs
*CYTB* ‐ 224	185 bp (12.76%)	50 (12.66%)	34 (13.18%)
*CYTB* ‐ 306	267 bp (18.41%)	89 (22.53%)	55 (21.32%)
*ND5* a	263 bp (18.14%)	72 (18.23%)	47 (18.22%)
*ND5* b	207 bp (14.28%)	60 (15.19%)	44 (17.05%)
*ND2*	423 bp (29.17%)	97 (24.56%)	65 (25.19%)
*COI*	105 bp (7.24%)	27 (6.84%)	13 (5.04%)
Final matrix	1450 bp (100%)	395 (100%)	258 (100%)

Abbreviations: bp, base pairs: PICs, parsimony‐informative characters.

* After trimming the primers.

### 3.3 Phylogeny and Molecular Species Delimitation

**FIGURE 3:**
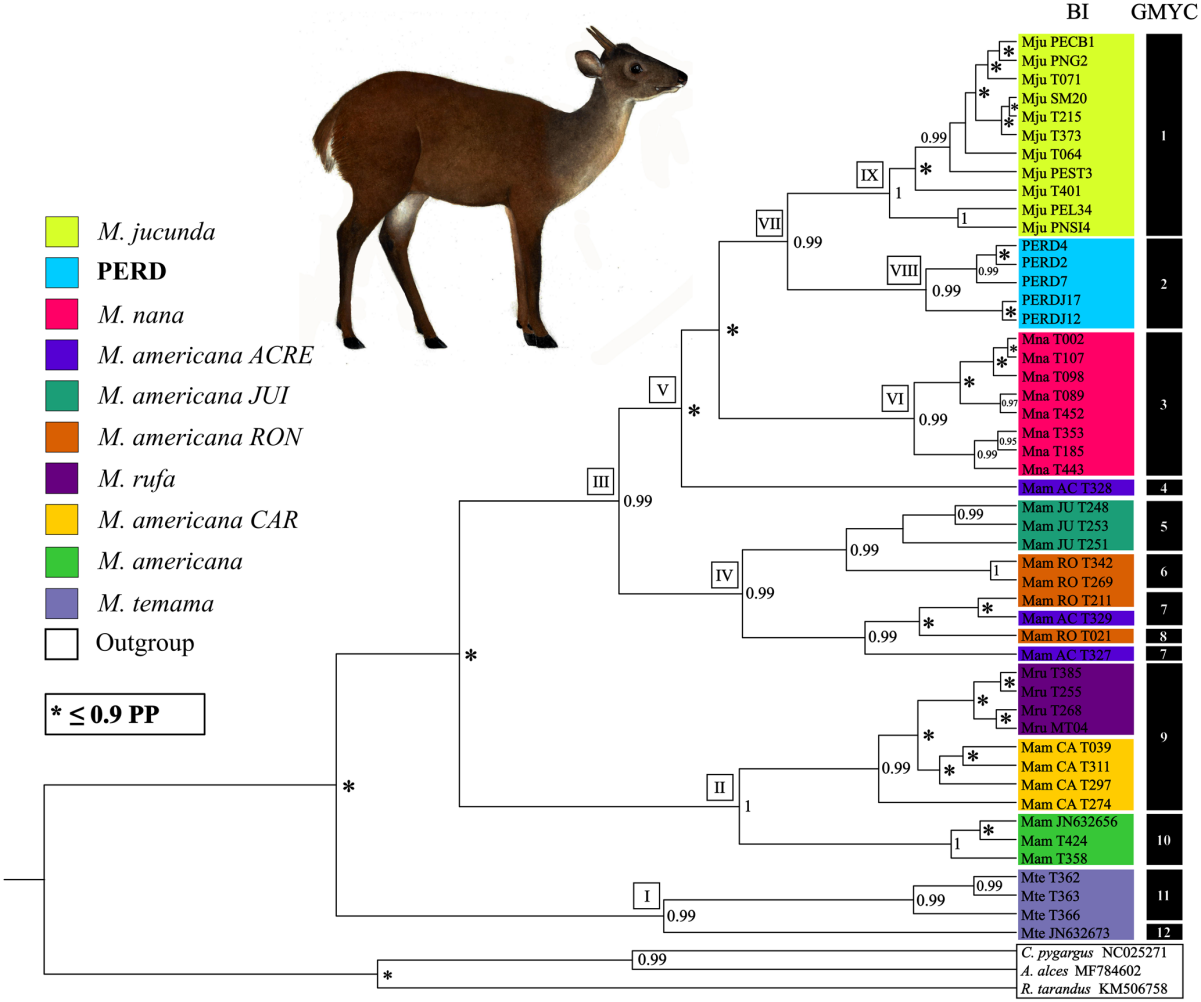
Ultrametric tree derived from the phylogenetic analysis with BEAST and species partitioning obtained using the *General Mixed Yule Coalescent* method (GMYC; single‐threshold, *p* = 0.001). The statistical support values of the clades are represented by posterior probability (PP). Roman numerals (I—IX; white boxes with black borders) identify the highlighted clades and Arabic numerals (1–12; black boxes) identify the MOTUs (molecular operational taxonomic units), both cited throughout the text. Samples labeled with “T” followed by three digits represent NUPECCE vouchers and samples labeled by eight digits correspond to sequences obtained from GenBank. Illustration source: Natalia de Azevedo (Azevedo et al. 2021).

The phylogenetic inference generated with BEAST successfully positioned the PERD specimens within the genus *Mazama* and identified them as the sister group to the reference *M. jucunda* , both of which were highly supported (PP > 0.9) (Figure 3). The analysis recovered three main clades in the *Mazama* topology. The first (clade I) includes samples of *M. temama* ; the second (clade II) contains samples of *M. americana* , *M. rufa* , and *M. americana* Carajás cytotype; and the third (clade III) includes samples of *M. americana* from the Acre, Juína, and Rondônia cytotypes, *M. nana* , *M. jucunda* , and the samples from PERD. Clade VII is noteworthy for grouping, in a reciprocally monophyletic way, the samples of PERD (clade VIII) and *M. jucunda* (clade IX).

The four molecular species delimitation analyses identified between 12 and 25 MOTUs. In the GMYC method, both single‐ and multiple‐threshold hypotheses were statistically significant, with *p* = 0.001 and 0.04, respectively. The single‐threshold hypothesis resulted in 12 MOTUs; and the multiple‐thresholds in 15 MOTUs. The analyses using the bPTP method revealed 18 MOTUs with the non‐ultrametric tree, compared to 25 MOTUs identified by the ultrametric tree (Figure S2). Here, we only present the results from the most conservative species delimitation approach considering the current literature, which was the GMYC single‐threshold hypothesis (Figure 3). This approach indicated two MOTUs for the clades representing PERD (clade VIII) and other *M. jucunda* (clade IX) samples. The other hypotheses produced more MOTUs (including single‐specimen groupings) and artificially split well‐established species like *M. nana* , *M. americana* , and *M. temama* . Complete results for all four tested hypotheses are available in Figure S2.

### 3.4 Haplotype Network and Genetic Distance

**FIGURE 4:**
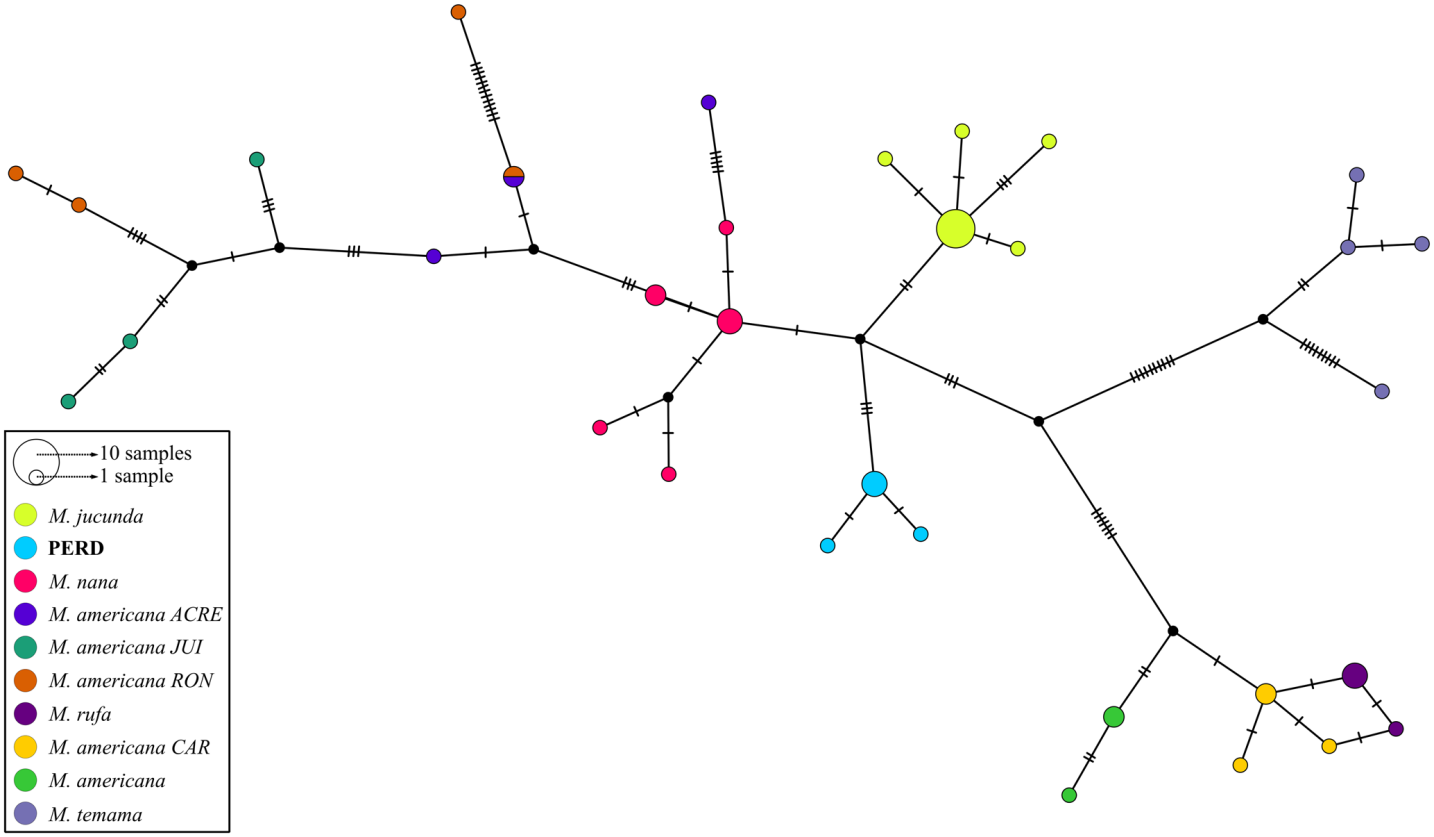
Haplotype network generated from the final matrix by median‐joining method. The traces on the branches represent mutational steps. PERD = Rio Doce State Park; ACRE = *M. americana* Acre cytotype; JUI = *M. americana* Juína cytotype; RON = *M. americana* Rondônia cytotype; CAR = *M. americana* Carajás cytotype.

The haplotype network (Figure 4) showed that the PERD population possesses unique and exclusive haplotypes which are not shared with any other species. In addition, we observed that five mutational steps separate the PERD haplotype group from that of *M. jucunda* , while three mutational steps separate *M. jucunda* and *M. nana* .

The smallest genetic distance between the PERD population and any other species was with *M. jucunda* (0.0158). This value is very close to the genetic distance between *M. jucunda* and its closest related species, *M. nana* (0.0165) (Table 2).

**TABLE 2:** Average pairwise genetic distance between species and the population of Rio Doce State Park (PERD).

Species	*Mju*	PERD	*Mna*	*MamAC*	*MamJU*	*MamRO*	*MamCA*	*Mru*	*Mam*
PERD	0.0158								
*Mna*	0.0165	0.0162							
*MamAC*	0.0226	0.0231	0.0158						
*MamJU*	0.0260	0.0231	0.0221	0.0189					
*MamRO*	0.0293	0.0292	0.0236	0.0205	0.0195				
*MamCA*	0.0472	0.0464	0.0382	0.0428	0.0497	0.0496			
*Mru*	0.0489	0.0477	0.0400	0.0429	0.0499	0.0503	0.0043		
*Mam*	0.0499	0.0495	0.0411	0.0450	0.0511	0.0513	0.0160	0.0173	
*Mte*	0.0593	0.0591	0.0558	0.0561	0.0583	0.0598	0.0616	0.0594	0.0562

*Note:* *Mju* = *M. jucunda* ; PERD = Rio Doce State Park; *Mna* = *M. nana* ; *MamAC* = *M. americana* Acre cytotype; *MamJU* = *M. americana* Juína cytotype; *MamRO* = *M. americana* Rondônia cytotype; *MamCA* = *M. americana* Carajás cytotype; *Mru* = *M. rufa* and *Mam* = *M. americana* .

## 4 Discussion

Our phylogenetic results linked the red brocket deer from PERD to *M. jucunda* rather than to any *M. americana* lineage. However, there was clear phylogenetic structuring with high statistical support, which also showed geographical coherence. PERD is situated in the state of Minas Gerais, whereas all other *M. jucunda* samples originated from southern areas over 700 km away, specifically from the states of São Paulo, Paraná, and Santa Catarina. The analyses of this population conducted by Oliveira et al. (2020) (530 bp of the *CYTB* gene) did not allow species identification and could only assign the population to the genus *Mazama*. In this study, we identified the PERD population as closely related to *M. jucunda* using six mitochondrial markers and an expanded sampling of both PERD samples and *M. jucunda* individuals across the species' known distribution.

The results of the species delimitation analysis (Figure 3) support the presence of genetic structure between the PERD population and the other *M. jucunda* samples. The GMYC single‐threshold approach was the most conservative among the methods tested (GMYC multiple‐thresholds; bPTP with ultrametric/non‐ultrametric trees), as it best aligned with the current taxonomic arrangement of the group (Merino and Rossi 2010; Peres, Grotta‐Neto, et al. 2021), the chromosome lineages identified for red brocket deer (Cursino et al. 2014; Salviano et al. 2017; Galindo et al. 2021; Bernegossi et al. 2024), and avoided artificial splitting of well‐established species, as suggested by the phylogenetic analysis. In general, the other approaches tested, especially those based on the bPTP method, showed excessive subdivision (Figure S2).

The discordance between the delimitation methods (GMYC vs. bPTP) reflects the lumper–splitter debate (Isaac et al. 2004), particularly acute in rapidly radiating taxa such as *Mazama* (Duarte et al. 2008). Given the congruence of the GMYC single‐threshold approach with established taxonomy and cytogenetic data, we favor this conservative delimitation unless robust integrative evidence (e.g., morphology, karyotypes) supports finer divisions. Although this approach may mask unique lineages, it avoids taxonomic inflation (Isaac et al. 2004).

The combined results from the haplotype network (Figure 4) and genetic distance matrix (Table 2) suggest limited or historically absent gene flow (from the maternal lineage, as only mtDNA was analyzed) between the PERD population and other *M. jucunda* populations, indicating potential genetic structuring. While these patterns could reflect species‐level divergence—given that the genetic distance between PERD and *M. jucunda* is comparable to that between *M. jucunda* and *M. nana* —this interpretation should be viewed with caution. Alternative explanations, such as historical isolation with subsequent genetic drift or sex‐biased dispersal (e.g., male‐mediated gene flow), are also plausible and warrant further investigation (e.g., using nuclear markers and broader sampling).

It is important to highlight that *M. nana* is the phylogenetically closest species to *M. jucunda* , being recovered as a sister species with reciprocal monophyly in this study (Figure 3) and in other studies (Duarte et al. 2008; Gutiérrez et al. 2017; Peres, Grotta‐Neto, et al. 2021). They were separated from a hypothetical common ancestor in the early Pleistocene, approximately 1 million years ago (Duarte et al. 2008), and are widely recognized from a taxonomic perspective (Abril et al. 2010; Vogliotti and Duarte 2010). Their morphological patterns are clearly differentiated (Rossi 2000), and their chromosomal patterns have already been described: *M. jucunda* has a diploid chromosome number (2n) = 32–34 and a fundamental number (FN) = 46 (Duarte and Jorge 2003; Vogliotti and Duarte 2010), whereas *M. nana* has 2n = 36–39 and FN = 58 (Abril et al. 2010; Abril and Duarte 2008). Such chromosomal differences represent an important mechanism of reproductive isolation, falling within the biological species concept (Cursino et al. 2014; Salviano et al. 2017; Galindo et al. 2021; Bernegossi et al. 2024).

While our molecular data reveal significant divergence between the PERD population and other *M. jucunda* lineages—comparable to differences between *M. jucunda* and *M. nana* —we emphasize that these results alone are not conclusive for species delimitation. The observed phylogeographic structure may reflect either incipient speciation or strongly differentiated populations. Chromosomal divergence, a known post‐zygotic barrier in *Mazama* (Cursino et al. 2014; Salviano et al. 2017), remains untested in PERD specimens. Given the reliance of the genus on cytogenetic data for taxonomic resolution (Jorge and Benirschke 1977; Duarte and Jorge 2003; Peres, Grotta‐Neto, et al. 2021), we stress the urgency of karyotypic analysis to determine whether PERD represents a distinct species (if chromosomal differences mirror *M. jucunda* – *M. nana* divergence) or an evolutionarily significant unit (ESU) within *M. jucunda* (if the chromosomes are conserved). Until such evidence is available, we conservatively classify PERD as an ESU of *M. jucunda* , acknowledging its genetic distinctiveness, while avoiding premature taxonomic inflation.

The ESU concept, first introduced by Ryder (1986), is generally defined as a population or group of populations that is considered distinct for conservation purposes due to its unique genetic makeup and evolutionary history (Ryder 1986), which was demonstrated in our study by the reciprocally monophyletic topology, isolation pattern in the haplotype network, and genetic distance. Identifying ESUs is crucial for developing effective conservation strategies, such as captive breeding, habitat protection, and translocation efforts, as it ensures that conservation actions maintain genetic diversity and evolutionary potential (Hutama et al. 2017; Forester et al. 2022). This concept has been applied to deer species (Zachos et al. 2014; Singh et al. 2021), including Neotropical species (Oliveira et al. 2020; Gonzalez et al. 2024).

The exclusive use of mitochondrial markers for genetic analysis and the absence of complementary data sources, such as cytogenetic, morphological, and ecological information, pose limitations to this study. Thus, the results should be interpreted with caution, as gene trees based on mitochondrial regions may overestimate structuring when there is female philopatry (Hoelzer 1997; Eberle et al. 2019). Despite this, mitochondrial markers have been widely used in phylogenetic studies of Neotropical deer in recent years (Gilbert et al. 2006; Duarte et al. 2008; Gutiérrez et al. 2015, 2017; Escobedo‐Morales et al. 2016, 2023; Heckeberg 2020; Cifuentes‐Rincón et al. 2020; Peres, Grotta‐Neto, et al. 2021; Bernegossi et al. 2023; Sandoval et al. 2022). Moreover, some studies have tested the use of nuclear markers, but they have not been informative for recovering phylogenetic relationships between lineages with recent diversification (Gilbert et al. 2006; Heckeberg 2020; Vozdova et al. 2021).

Finally, it is worth clarifying that the PCR‐RFLP protocol, which in theory would allow the identification of samples of *Mazama* species from the Atlantic Forest (González et al. 2009), was not able to achieve species‐level identification in this study because PERD samples have a mutation at the enzyme cleavage site that would discriminate *M. jucunda* from *M. nana* . The same mutation was also observed in samples previously identified as *M. jucunda* , for example, animal T401 from Campo Alegre, SC (Table S2), and samples PEL34 and PNSI4 from Lauráceas State Park, PR, and Serra do Itajaí National Park, SC, respectively (Table S3). Such a mutation has already been reported (Grotta‐Neto 2020) and highlights the need for caution when applying the PCR‐RFLP method for species‐specific identification of samples, considering that this approach is limited to the reference samples used as a comparative basis to establish the enzymatic cleavage sites. Further studies should review this protocol and include a more representative group of reference samples and new enzyme sites.

## 5 Conclusion Remarks and Conservation Implications

In this study, we report a new population of the small red brocket deer (*Mazama jucunda*) in the Rio Doce State Park (PERD), marking the first record for the state of Minas Gerais, which is more than 700 km from the species' known geographical distribution (Vogliotti et al. 2016; Duarte et al. 2017). However, the genetic structure observed in the analyses, along with the geographic isolation of PERD, suggests that this population represents an evolutionarily significant unit (ESU), and it is possible that it is indeed a distinct species.

Despite some limitations, the present study represents an important advance in the knowledge of the geographical distribution of Neotropical forest deer species, especially regarding *M. jucunda* , the largest endemic animal species in the Atlantic Forest, which is one of the main biodiversity hotspots globally (Myers et al. 2000). In the short term, the most pressing conservation implication for this species is likely to be a change in the assessment of extinction risk. Currently, *M. jucunda* is classified as Vulnerable by the IUCN under criterion C2aii (Vogliotti et al. 2016), meaning that the species has an estimated population of fewer than 10 000 individuals, is undergoing a decline, and all mature individuals are found in a single subpopulation (IUCN 2012). Including the PERD population, the species has two subpopulations and therefore may have a larger population size; thus, *M. jucunda* would no longer be Vulnerable. Meanwhile, owing to its ESU status, management actions should be customized for this population separately from the population in the south. Future research should aim to capture individuals to perform a thorough characterization by analyzing morphological, molecular, and cytogenetic data. This will allow us to confirm whether the population represents only an ESU or whether it is a new species. In the case of a new species, the description of its geographic distribution should be evaluated in future studies.

## Author Contributions

**Jeferson L. S. Freitas:** conceptualization, investigation, formal analysis, writing – original draft, writing – review and editing, funding acquisition. **Pedro H. F. Peres:** investigation, formal analysis, writing – review and editing. **Francisco Grotta‐Neto and Márcio L. Oliveira:** investigation, writing – review and editing. **José M. B. Duarte:** conceptualization, supervision, project Administration, writing – review and editing, funding acquisition.

## Funding

This work was supported by the São Paulo Research Foundation (FAPESP; no. 2021/02087‐2 and 2017/07014‐8) and the Coordination for the Improvement of Higher Education Personnel (CAPES; no. 88887.597519/2021‐00).

## Ethics Statement

All data collection in this study was approved by the competent environmental agencies under authorizations IEF‐MG no. 015/2021 and SISBIO no. 77 907.

## Conflicts of Interest

The authors declare no conflicts of interest.

## Supporting information


**Figure S1:** Reference and fecal samples included in this study. For specific locations, please refer to Tables S2 and S3.
**Figure S2:** (A) Ultrametric tree derived from the phylogenetic analysis using BEAST. (B–E) Molecular species delimitation by the General Mixed Yule Coalescent (GMYC) method, (B) single‐threshold (*p* = 0.001) and (C) multiple‐thresholds (*p* = 0.04); and by the Bayesian implementation of the Poisson Tree Process (bPTP) method, (D) non‐ultrametric tree and (E) ultrametric tree. The statistical support values for the clades are represented by posterior probability (PP). Numbers in Arabic numerals (1–25; black and grayscale boxes) identify the MOTUs (molecular operational taxonomic units). Samples labeled with “T” followed by three digits represent NUPECCE vouchers and samples labeled by eight digits correspond to sequences obtained from GenBank.
**Table S1:** Features of the primers used to amplify mitochondrial DNA from fecal samples. bp = base pairs.
**Table S2:** Specimens and sequences included in the reference dataset for the phylogenetic analysis. Each sample is categorized as belonging to either the ingroup matrix or the outgroup matrix. The source of the sequences is indicated by the GenBank accession number. PE = State Park. Brazilian states: AC = Acre, GO = Goiás, MA = Maranhão, MT = Mato Grosso, PA = Pará, PR = Paraná, RO = Rondônia, RR = Roraima, RS = Rio Grande do Sul, SC = Santa Catarina, SP = São Paulo.
**Table S3:** Samples and sequences included in the fecal dataset for phylogenetic analysis. Each sample is categorized as originating from either the fecal database of NUPECCE (“bank”) or from this study (“this study”). The source of the sequences is indicated by the GenBank accession number. PE = State Park, PN = National Park, RPPN = Natural Heritage Private Reserve. Brazilian states: MG = Minas Gerais, PR = Paraná, SP = São Paulo, SC = Santa Catarina.

## Data Availability

The data produced in this study are available in GenBank. The access codes are listed in the Tables S2 and S3.
