# Supplementary material for: Fecal DNA Identifies a Disjunct Population of an Endemic Deer (Mazama jucunda) From the Atlantic Forest, Brazil
Source: Anim Genet. 2026 Jun 5;57(3):e70134. doi: 10.1002/age.70134 (PMC13241587; doi:10.1002/age.70134)
Supplement: Supplementary file 1 — Figure S1: Reference and fecal samples included in this study. For specific locations, please refer to Tables S2 and S3. Figure S2: (A) Ultrametric tree derived from the phylogenetic analysis using BEAST. (B–E) Molecular species delimitation by the General Mixed Yule Coalescent (GMYC) method, (B) single‐threshold (p = 0.001) and (C) multiple‐thresholds (p = 0.04); and by the Bayesian implementation of the Poisson Tree Process (bPTP) method, (D) non‐ultrametric tree and (E) ultrametric tree. The statistical support values for the clades are represented by posterior probability (PP). Numbers in Arabic numerals (1–25; black and grayscale boxes) identify the MOTUs (molecular operational taxonomic units). Samples labeled with “T” followed by three digits represent NUPECCE vouchers and samples labeled by eight digits correspond to sequences obtained from GenBank. Table S1: Features of the primers used to amplify mitochondrial DNA from fecal samples. bp = base pairs. Table S2: Specimens and sequences included in the reference dataset for the phylogenetic analysis. Each sample is categorized as belonging to either the ingroup matrix or the outgroup matrix. The source of the sequences is indicated by the GenBank accession number. PE = State Park. Brazilian states: AC = Acre, GO = Goiás, MA = Maranhão, MT = Mato Grosso, PA = Pará, PR = Paraná, RO = Rondônia, RR = Roraima, RS = Rio Grande do Sul, SC = Santa Catarina, SP = São Paulo. Table S3: Samples and sequences included in the fecal dataset for phylogenetic analysis. Each sample is categorized as originating from either the fecal database of NUPECCE (“bank”) or from this study (“this study”). The source of the sequences is indicated by the GenBank accession number. PE = State Park, PN = National Park, RPPN = Natural Heritage Private Reserve. Brazilian states: MG = Minas Gerais, PR = Paraná, SP = São Paulo, SC = Santa Catarina. [file AGE-57-0-s001.docx]

**Supplementary information**

**Article title**: Fecal DNA identifies a disjunct population of an endemic deer (*Mazama jucunda*) from the Atlantic Forest, Brazil.

**Journal name**: Animal Genetics.

**Author names**:

Jeferson L. S. Freitas^1,2^, Pedro H. F. Peres^1^, Francisco Grotta-Neto^1^, Márcio L. Oliveira^3^, José M. B. Duarte^1^

**Corresponding authors**: jeferson.freitas.bio@gmail.com, mauricio.barbanti@unesp.br

**Affiliations**:

^1^Deer Research and Conservation Center (NUPECCE), São Paulo State University (UNESP), Jaboticabal, SP, Brazil

^2^Evolutionary Genetics and Molecular Biology Graduate Program, Federal University of São Carlos (UFSCar), São Carlos, SP, Brazil

^3^Department of Biological Sciences and Health, University of Araraquara, Araraquara (UNIARA), SP, Brazil


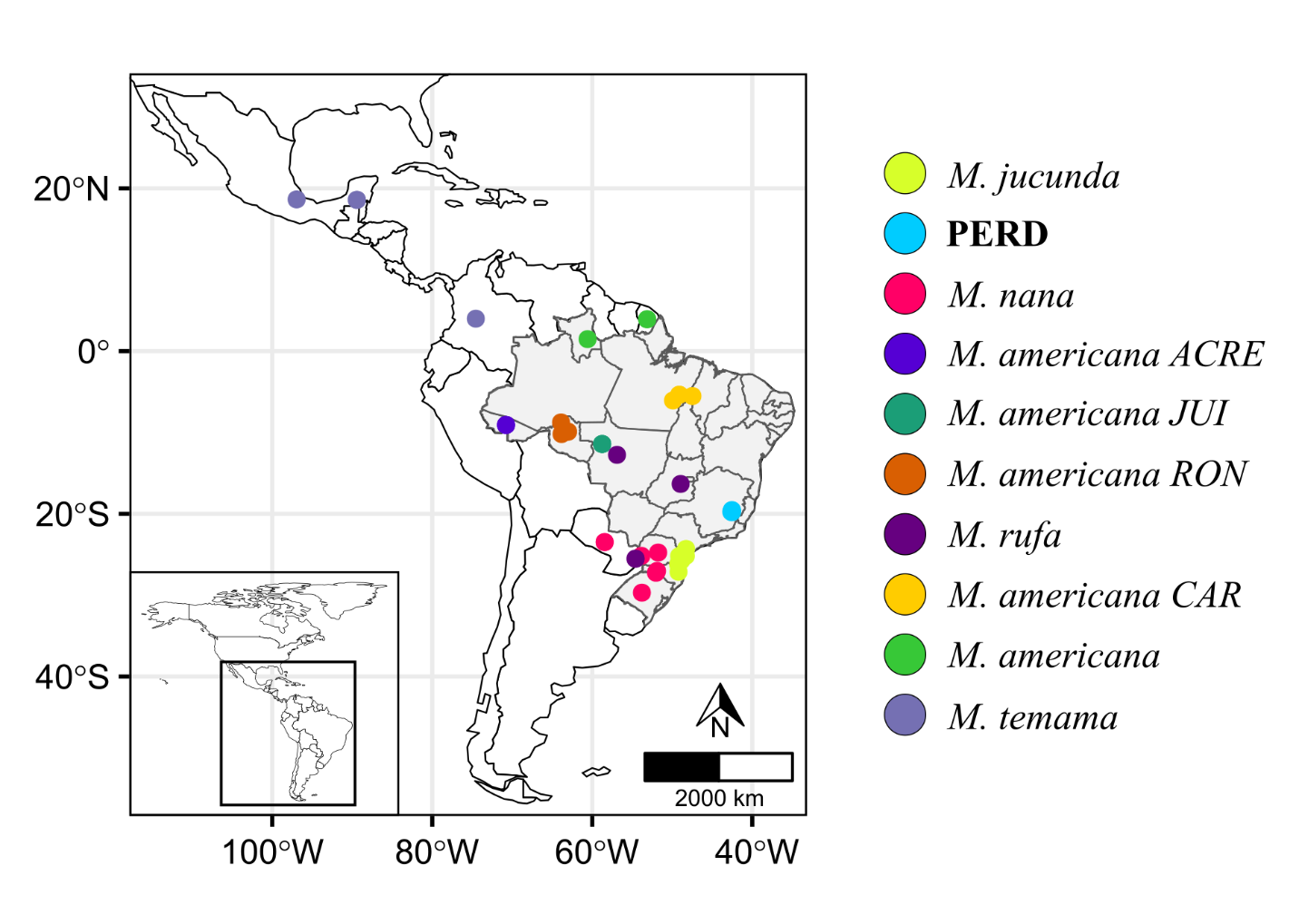


**Fig. S1** Reference and fecal samples included in this study. For specific locations, please refer to Tables S2 and S3.


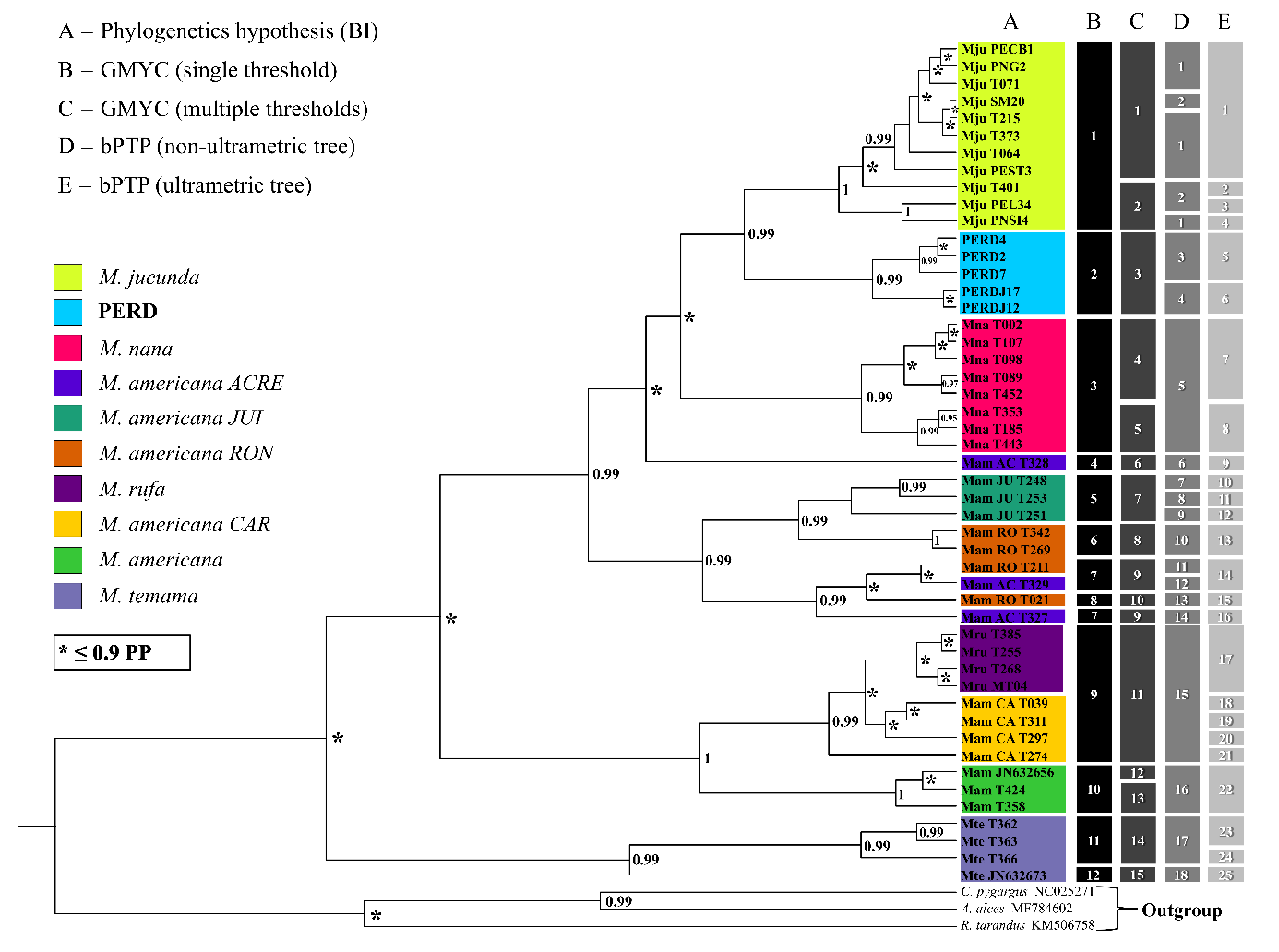


**Fig. S2** (A) Ultrametric tree derived from the phylogenetic analysis using BEAST. (B-E) Molecular species delimitation by the General Mixed Yule Coalescent (GMYC) method, (B) single-threshold (p = 0.001) and (C) multiple-thresholds (p = 0.04); and by the Bayesian implementation of the Poisson Tree Process (bPTP) method, (D) non-ultrametric tree and (E) ultrametric tree. The statistical support values for the clades are represented by posterior probability (PP). Numbers in Arabic numerals (1 – 25; black and grayscale boxes) identify the MOTUs (molecular operational taxonomic units). Samples labeled with “T” followed by three digits represent NUPECCE vouchers and samples labeled by eight digits correspond to sequences obtained from GenBank.

**Table S1** Features of the primers used to amplify mitochondrial DNA from fecal samples. bp = base pairs.

| **Region** | **Size** | **Id** | **Primer (5’ – 3’)** | **Source** |
| --- | --- | --- | --- | --- |
| *CYTB* | 224 bp | IDMAZ 224 L | CATCCGACACAATAACAGCA | González et al., 2009 |
|  |  | IDMAZ H | TCCTACGAATGCTGTGGCTA |  |
| *CYTB* | 306 bp | CytB 306 F | CCATCAGACGCAGACAAA | Leandro et al. (2022) |
|  |  | CytB 306 R | GCGTTGTTTAGATGTGTGAAG |  |
| *ND5* | 305 bp | ND5a F Fec | TCTCAAACTGACACTGACTTAC | Peres, 2020 |
|  |  | ND5a R Fec | TGCATCTGTTCGTCCATATC |  |
| *ND5* | 249 bp | ND5b F Fec | CCAACCCTACCTAGCATTTCTC | Peres, 2020 |
|  |  | ND5b R Fec | TACGACGTATTGGCGGTTTC |  |
| *ND2* | 463 bp | ND2A F | CATACCCCGAAAATGTTGGT | Ricoldi, 2021 |
|  |  | ND2A R | GTACGGATATGGGTGCCAGT |  |
| *COI* | 149 bp | COI 149 F | GTGCTCCAGATATAGCATTTCC | González, 2023* |
|  |  | COI 149 R | GCTAGATTGCCAGCTAAAGGG |  |

*Personal communication.

**Table S2** Specimens and sequences included in the reference dataset for the phylogenetic analysis. Each sample is categorized as belonging to either the ingroup matrix or the outgroup matrix. The source of the sequences is indicated by the GenBank accession number. PE = State Park. Brazilian states: AC = Acre, GO = Goiás, MA = Maranhão, MT = Mato Grosso, PA = Pará, PR = Paraná, RO = Rondônia, RR = Roraima, RS = Rio Grande do Sul, SC = Santa Catarina, SP = São Paulo.

| **Species** | **NUPECCE Voucher** | **Cytotype** | **Locality** | **Matrix** | **Sequence source** | | | | | |
| --- | --- | --- | --- | --- | --- | --- | --- | --- | --- | --- |
|  |  |  |  |  | **CYTB^1^** | **CYTB^2^** | **ND5^1^** | **ND5^2^** | **ND2** | **COI** |
| *M. americana* | T358 | - | French Guiana | Ingroup | MZ350857 | MZ350857 | MZ350857 | MZ350857 | MZ350857 | MZ350857 |
| *M. americana* | T424 | - | Brazil, RR | Ingroup | PV350845 | PV350874 | PV350988 | PV350950 | PV350909 | PV300000 |
| *M. americana* | - | - | French Guiana | Ingroup | JN632656 | JN632656 | JN632656 | JN632656 | JN632656 | JN632656 |
| *M. americana* | T327 | Acre | Brazil, AC | Ingroup | PV350860 | PV350899 | PV351016 | PV350973 | PV350934 | PV299975 |
| *M. americana* | T328 | Acre | Brazil, AC | Ingroup | PV350871 | PV350907 | PV351014 | PV350986 | PV350944 | PV299979 |
| *M. americana* | T329 | Acre | Brazil, AC | Ingroup | PV350861 | PV350891 | PV351015 | PV350972 | PV350935 | PV299976 |
| *M. americana* | T039 | Carajás | Brazil, PA  Paraupebas | Ingroup | PV350840 | PV350876 | PV350994 | PV350956 | PV350914 | PV299995 |
| *M. americana* | T274 | Carajás | Brazil, MA  Imperatriz | Ingroup | PV350842 | PV350879 | PV350990 | PV350953 | PV350910 | PV299993 |
| *M. americana* | T297 | Carajás | Brazil, PA  Marabá | Ingroup | OQ731411 | OQ731411 | OQ731411 | OQ731411 | OQ731411 | OQ731411 |
| *M. americana* | T311 | Carajás | Brazil, PA  Marabá | Ingroup | PV350841 | PV350877 | PV350993 | PV350955 | PV350915 | PV299996 |
| *M. americana* | T248 | Juína | Brazil, MT  Juína | Ingroup | PV350858 | PV350902 | PV351019 | PV350983 | PV350945 | PV299972 |
| *M. americana* | T251 | Juína | Brazil, MT  Juína | Ingroup | PV350855 | PV350901 | PV351018 | PV350982 | PV350946 | PV299971 |
| *M. americana* | T253 | Juína | Brazil, MT  Juína | Ingroup | MZ350856 | MZ350856 | MZ350856 | MZ350856 | MZ350856 | MZ350856 |
| *M. americana* | T021 | Rondônia | Brazil, RO  Ariquemes | Ingroup | DQ789216 | DQ789216 | MZ488895 | MZ488895 | PV350932 | PV299970 |
| *M. americana* | T211 | Rondônia | Brazil, RO  Ariquemes | Ingroup | PV350859 | PV350890 | PV351017 | PV350971 | PV350933 | PV299974 |
| *M. americana* | T269 | Rondônia | Brazil, RO  Buritis | Ingroup | PV350856 | PV350903 | PV351020 | PV350984 | PV350947 | PV299977 |
| *M. americana* | T342 | Rondônia | Brazil, RO  Porto Velho | Ingroup | PV350857 | PV350904 | PV351021 | PV350985 | PV350948 | PV299978 |
| *M. jucunda* | T064 | - | Brazil, PR | Ingroup | DQ789228 | DQ789228 | MZ488898 | MZ488898 | PV350923 | PV299984 |
| *M. jucunda* | T071 | - | Brazil, PR  Barra do Turvo | Ingroup | PV350865 | PV350886 | PV351001 | PV350961 | PV350920 | PV299985 |
| *M. jucunda* | T215 | - | Brazil, SP  PE Intervales | Ingroup | PV350866 | PV350887 | PV350997 | PV350963 | PV350921 | PV299986 |
| *M. jucunda* | T373 | - | Brazil, PR  Bocaúva do Sul | Ingroup | PV350867 | PV350888 | PV350998 | PV350964 | PV350922 | PV299988 |
| *M. jucunda* | T401 | - | Brazil, SC  Campo Alegre | Ingroup | PV350869 | PV350892 | PV350996 | PV350962 | PV350926 | PV299987 |
| *M. nana* | T002 | - | Paraguai | Ingroup | DQ789214 | DQ789214 | MZ488902 | MZ488902 | PV350936 | PV300001 |
| *M. nana* | T089 | - | Brazil, RS  Santa Maria | Ingroup | PV350848 | PV350896 | PV351007 | PV350975 | PV350937 | PV300002 |
| *M. nana* | T098 | - | Brazil, SC  Concordia | Ingroup | PV350849 | PV350893 | PV351008 | PV350976 | PV350938 | PV300003 |
| *M. nana* | T107 | - | Paraguai | Ingroup | PV350850 | PV350895 | PV351010 | PV350977 | PV350939 | PV300005 |
| *M. nana* | T185 | - | Brazil, PR  Céu Azul | Ingroup | PV350853 | PV350894 | PV351009 | PV350981 | PV350941 | PV300004 |
| *M. nana* | T353 | - | Brazil, PR  Pitanga | Ingroup | PV350854 | PV350897 | PV351013 | PV350980 | PV350942 | PV300007 |
| *M. nana* | T443 | - | Brazil, SC  Concórdia | Ingroup | PV350852 | PV350898 | PV351012 | PV350979 | PV350943 | PV300008 |
| *M. nana* | T452 | - | Brazil, SC  Irani | Ingroup | PV350851 | PV350900 | PV351011 | PV350978 | PV350940 | PV300006 |
| *M. rufa* | MT04 | - | Brazil, MT | Ingroup | PV350839 | PV350875 | PV350989 | PV350951 | PV350911 | PV299994 |
| *M. rufa* | T255 | - | Brazil, GO  Anápolis | Ingroup | PV350844 | PV350880 | PV350991 | PV350952 | PV350912 | PV299997 |
| *M. rufa* | T268 | - | Brazil, PR  Foz do Iguaçú | Ingroup | PV350843 | PV350878 | PV350992 | PV350954 | PV350913 | PV299998 |
| *M. rufa* | T385 | - | Brazil, PR  Foz do Iguaçú | Ingroup | OQ198444 | OQ198444 | OQ198444 | OQ198444 | OQ198444 | OQ198444 |
| *M. temama* | T366 | - | Mexico | Ingroup | MZ350864 | MZ350864 | MZ350864 | MZ350864 | MZ350864 | MZ350864 |
| *M. temama* | T362 | - | Mexico | Ingroup | MZ362858 | MZ362858 | MZ362858 | MZ362858 | MZ362858 | MZ362858 |
| *M. temama* | T363 | - | Mexico | Ingroup | PV350872 | PV350908 | PV351024 | PV350987 | PV350949 | PV299969 |
| *M. temama* | - | - | Colômbia | Ingroup | JN632673 | JN632673 | JN632673 | JN632673 | JN632673 | JN632673 |
| *Alces alces* | - | - | - | Outgroup | MF784602 | MF784602 | MF784602 | MF784602 | MF784602 | MF784602 |
| *Capreolus pygargus* | - | - | - | Outgroup | NC025271 | NC025271 | NC025271 | NC025271 | NC025271 | NC025271 |
| *Rangifer tarandus* | - | - | - | Outgroup | KM506758 | KM506758 | KM506758 | KM506758 | KM506758 | KM506758 |

**Table S3** Samples and sequences included in the fecal dataset for phylogenetic analysis. Each sample is categorized as originating from either the fecal database of NUPECCE (“bank”) or from this study (“this study”). The source of the sequences is indicated by the GenBank accession number. PE = State Park, PN = National Park, RPPN = Natural Heritage Private Reserve. Brazilian states: MG = Minas Gerais, PR = Paraná, SP = São Paulo, SC = Santa Catarina.

| **Id** | **Locality** | **Species** | **Sampling** | **State** | **Sequence source** | | | | | |
| --- | --- | --- | --- | --- | --- | --- | --- | --- | --- | --- |
|  |  |  |  |  | **CYTB^1^** | **CYTB^2^** | **ND5^1^** | **ND5^2^** | **ND2** | **COI** |
| PERD2 | PE Rio Doce | - | Bank | MG | MT008200 | MT008228 | PV351006 | PV350966 | PV350930 | PV299980 |
| PERD4 | PE Rio Doce | - | Bank | MG | MT008201 | MT008229 | PV351002 | PV350974 | PV350931 | PV299981 |
| PERD7 | PE Rio Doce | - | Bank | MG | MT008203 | MT008231 | PV351005 | PV350967 | PV350927 | PV299982 |
| PERDJ12 | PE Rio Doce | - | This study | MG | PV350846 | PV350905 | PV351004 | PV350968 | PV350928 | PV299983 |
| PERDJ17 | PE Rio Doce | - | This study | MG | PV350847 | PV350906 | PV351003 | PV350969 | PV350929 | NA |
| PECB1 | PE Carlos Botelho | *M. jucunda* | Bank | SP | PV350862 | PV350881 | PV351000 | PV350957 | PV350916 | PV299973 |
| PEL34 | PE Lauráceas | *M. jucunda* | Bank | PR | PV350868 | PV350889 | PV351025 | PV350958 | PV350925 | PV299999 |
| PEST3 | PE Serra do Tabuleiro | *M. jucunda* | Bank | SC | PV350873 | PV350885 | PV350999 | PV350970 | PV350917 | PV299989 |
| PNG2 | PN Guaricana | *M. jucunda* | Bank | PR | PV350864 | PV350882 | PV351022 | PV350959 | PV350918 | PV299990 |
| PNSI4 | PN Serra do Itajaí | *M. jucunda* | Bank | SC | PV350870 | PV350884 | PV351023 | PV350965 | PV350924 | PV299992 |
| SM20 | RPPN Salto Morato | *M. jucunda* | Bank | PR | PV350863 | PV350883 | PV350995 | PV350960 | PV350919 | PV299991 |
